# Unsuspected Thoracic Aneurism

**Published:** 1895-08-10

**Authors:** Arthur Maude

**Affiliations:** Westerham


### Medical Progress and Hospital Clinics.

[*The Editor will be glad to receive offers of co-operation and contributions from members of the profession. All letters should be addressed to* The Editor, The Lodge, Porchester Square, London, W.]

### UNSUSPECTED THORACIC ANEURISM.

By Arthur Maude, Westerham.

The rupture of an unsuspected aneurism of the thoracic aorta is an occurrence by no means infrequent, and one so tragic to all concerned that we may well devote some attention to the subject.

The following cases have occurred recently in my practice:—

I. C. C., 56, plumber.—On April 2nd, 1891, rose as usual, ate a good breakfast, complained of slight difficulty in breathing, had a sudden large hæmoptysis, and died at once. He had always been in fair health, but had occasionally coughed up streaks of blood, and been at times somewhat short-winded, but had never been under medical treatment. I made a post-mortem examination. The ascending and transverse arch of the aorta was enormously enlarged, forming a conical pouch directed backwards, large enough (when emptied) to take my whole fist; it was filled with a hard, india-rubber-like, laminated clot of a blue-grey colour. The tumour pressed laterally on the trachea, which was flattened and reduced in calibre, and bent sideways over the aneurism. The rings of the trachea were much atrophied. The aorta was very atheromatous, with numerous calcareous plates. There was a small ragged opening from the left and posterior aspect of the aneurism into the trachea.

II. C. B., 44, joiner, had made no complaint of ill-health, and had not been under treatment. Was found dead in bed, February 12th, 1893, in a position of natural sleep. Post-mortem (by Mr. Maude): Body well nourished, muscular. Pericardium filled with about two quarts of soft dark clot; general hypertrophy of heart. Aorta very atheromatous, with numerous large calcareous patches. Cardiac valves all healthy. From the posterior aspect of the arch of the aorta is a ragged transverse opening 2 c.m. long, connecting the pericardium and a small sacculated aneurism, which had dissected between the walls of the aorta; it was filled by firm laminated clot.

III. A. B., 48, a retired cavalry officer. Appeared to have enjoyed excellent health; had never complained, or been under treatment or examination. On March 31st, 1895, he vaulted twice over a gate; he weighed fifteen or sixteen stone. Complained that day of some discomfort about the epigastrium, which increased next day, when he took lunch alone, and was found in the middle of the meal dead, reclining in a natural position in his chair.

P. M. (by Mr. Maude) showed the pericardium filled with two to three pints of clotted blood, heart normal in size for body weight, healthy in substance and valves. There was a uniform (fusiform) dilatation of the aorta immediately above the valves, but the amount of dilatation was not great. From the valvular ring in the posterior longitudinal axis of the vessel was a ragged split about 2 c.m. in length: aorta very thin and soft; all other organs healthy. He always seemed a remarkably healthy youthful man for his age, was very muscular and active, and had not been exposed to any of the factors which usually produce tissue degeneration. He had been in the cavalry about 30 years, had seen much active service, and had only just retired.

These cases illustrate many points of interest: "Rupture is by far the most common termination of aneurism, and the more latent the aneurism the more uniformly does death occur in this way."[1] "It will be observed," said Sibson, "that the greater the proportion of ruptures in any group of aneurisms of the aorta, the smaller is the proportion of instances in which the disease is indicated by symptoms during life, and *vice versâ*."[2]

As in my first case enormous dilatations may exist unsuspected, because no distress or incapacity is produced, incredible as this may seem. Quite recently Dr. Draper[3] has presented ten such cases of latent thoracic aneurism, and among them three were large enough to have produced erosion of vertebræ (without diagnosis).

Among other conclusions, which my cases quite corroborate, Draper claims that violent exertion is not necessary for the production of rupture; my first two cases support this assumption. In my third case the inference is unavoidable that rupture occurred the day before, just twenty-four hours before death, and that a slow leakage had continued into the pericardium. The clot formed was evidently partly "ante-mortem" clot; it was firm and light coloured, and occupied the posterior half of the cavity, as if the blood had slowly coagulated during the previous night.

Death, though sudden, is not necessarily instantaneous, and may be quite painless; the position of the bodies (in cases 2 and 3) in attitudes of natural repose leads to this conclusion, while it is impossible to suppose that such large quantities of blood as occupied the pericardium could have been forced into that cavity at the moment of a suddenly fatal rupture. The strong elastic structure of the pericardium could only permit a gradual distention, and must prevent a gush of blood so great as to kill by shock. Death by shock was, however, clearly the end of my first patient, for the amount of blood inhaled into the lung was extremely small, proving the instantaneous mode of death. In other cases the rupture may perhaps be only partial at first, permitting a slow leakage, and become extended later by stretching of the pericardium in the neighbourhood of its attachment round the aorta, and consequently of the aorta itself. The annals of punctured wounds of the heart afford analogies which help us here; for in the case of wounds the precise moment of first solution of the tissue is known, and the patients have survived many hours, or even days, to succumb ultimately to intra-pericardial hæmorrhage.[4]

My first two cases show how small a protection even a firm laminated clot of old standing affords against rupture of the wall; but it is noticeable that in neither case was the clot at all adherent to the endothelial lining.

My last case calls to mind the old-established observation of the great prevalence of aortic aneurism among soldiers, cavalry soldiers in particular. The army medical report of 1866[5] shows that death from this affection is eleven times more frequent than in the civil population. The assigned reason is, of course, tight dress and heavy acccoutrements worn during great and prolonged exertion. In the matter of accoutrements, the infantry officer enjoys a better position, as compared with his men, than does his cavalry colleague.

The only symptom which had attracted any attention in case I. was occasional slight blood spitting. His relatives were positive that this was in the form of mere streaks accompanying cough. We may, therefore, reject the idea that it was due to any leakage from the sac. The lungs presented after death nothing more remarkable than a little emphysema, but the pressure on the trachea, which was considerable, was fully sufficient to cause local congestion with slight staining of the sputa.

Hæmoptysis in aortic aneurism may be produced in various ways: (1) *direct* by leakage from the sac, (2) *indirect* by pressure (*a*) on the air passages, (*b*) on the lung substance, (*c*) on the blood vessels, (*d*) by interference with pulmonary circulation due to secondary valvular incompetence. In any very obscure case of hæmoptysis with scanty sputa occurring in a male, of middle or advanced age, who complains of vague cardiac symptoms, we ought to bear clearly in mind the possibility of thoracic aneurism.[6]

[1] R. Douglas Powell. Reynolds' System, Vol. V., p. 44. [2] The Aorta and the Aneurisms of the Aorta [3] Boston Med. and Surg. Journal, March 14, 1895. [4] Fischer. Langenbeck's Archives, Bd. IX., 1168. [5] Myers. On the Œtiology and Prevalence of Disease of the Heart Among Soldiers. 1870. Aitken. Scienc. and Practice of Medicine II., p. 644. Sixth Ed., 1872. [6] Gilbert Smithe Trans. Med. Soc., p. 324, XVI., 1893.

